# Bacterial Community Assembly Patterns Across Distinct Freshwater Habitats

**DOI:** 10.3390/biology15181609

**Published:** 2026-09-11

**Authors:** Shengnan Li, Zhe Wang, Xinyu Xie, Xun Xu, Min Wang, Ting Yi, Zhongyuan Shen, Ping Wu, Qianhong Gu

**Affiliations:** Engineering Research Center of Polyploid Fish Reproduction and Breeding of the State Education Ministry, College of Life Sciences, Hunan Normal University, Changsha 410081, China; 13848401469@163.com (Z.W.); xiexinyu317121@163.com (X.X.); 13837703742@163.com (X.X.); 202001094@hunnu.edu.cn (M.W.); yiting@hunnu.edu.cn (T.Y.); zyshen@hunnu.edu.cn (Z.S.); wuping@hunnu.edu.cn (P.W.); gqh@hunnu.edu.cn (Q.G.)

**Keywords:** community assembly, iCAMP, phylogenetic bins, freshwater, bacteria, 16S rRNA gene sequencing

## Abstract

Understanding microbial community assembly mechanisms in aquatic habitats is fundamental to predicting ecosystem responses to environmental change, yet systematic comparisons of deterministic versus stochastic process contributions among different water types remain limited. We conducted monthly sampling over one year from four freshwater habitats, including two aquaculture ponds (WC01, WC02), an enclosed urban lake (TZ), and a flowing river (XJ), and applied a phylogenetic-bin-based null model framework to uncover how bacterial community assembly processes change among habitats and time/season. The results indicated that homogeneous selection (33.5%), dispersal limitation (31.0%), and drift (26.5%) jointly governed community assembly across all samples. Among the four investigated systems, water type, rather than season or their interactions, emerged as the primary factor regulating assembly process differentiation. Specifically, homogeneous selection was significantly stronger in the two aquaculture ponds (WC01, WC02) than in the natural water bodies (TZ and XJ), while the flowing river XJ exhibited the highest dispersal limitation and the lowest drift. At the phylogenetic bin level, over 98% of bins switched their dominant assembly strategies across the four water bodies, especially between the two aquaculture ponds and the two natural water bodies. Environmental factor analyses also revealed habitat-specific driving patterns: nitrogen and phosphorus nutrients dominated homogeneous selection in the aquaculture ponds, whereas dissolved oxygen, turbidity and oxidation reduction potential mainly regulated dispersal limitation in the natural waters. Collectively, these findings reveal a hierarchical pattern of freshwater bacterial assembly with multi-process coordination, habitat dominance, and lineage-level differentiation, and underscore that lineage-level analyses are essential for uncovering assembly patterns hidden at the community level, offering practical guidance for microbial management under diverse hydrological conditions.

## Simple Summary

We collected water samples monthly over a full year from four typical freshwater habitats, including two aquaculture ponds, an urban lake and a river, to explore bacterial community assembly patterns across distinct habitats. We found that the inherent environment conditions of each water habitat, rather than seasonal changes, mainly determined the overall composition and assembly patterns of the bacterial community. The eutrophic aquaculture ponds rely on consistent environmental filtering to screen suitable bacteria, while flowing rivers are restricted by space and time barriers that hinder bacterial exchange. More importantly, different bacterial taxa show different assembly patterns in different water environments: only a few bacterial types can adapt well to all water habitats, and most bacteria will switch their assembly patterns when living in aquaculture ponds versus rivers or lakes. Changes in water temperature and conductivity act as universal factors that regulate the balance between consistent environmental screening and dispersal limitation. Our findings highlight the importance of taxa-level analyses for uncovering assembly patterns hidden at the community level and provide practical guidance for microbial management under diverse hydrological conditions.

## 1. Introduction

Disentangling the mechanisms that govern community assembly represent a central topic in ecology [1]. According to niche theory, deterministic processes, including environmental filtering, interspecific competition and facilitation, largely determine species composition and distribution. In contrast, neutral theory assumes ecological equivalence among species and emphasizes the role of stochastic processes such as birth, death, migration, speciation, and dispersal limitation in shaping communities [2]. Following decades of debate and synthesis, it is now broadly accepted that deterministic and stochastic process jointly shape local communities [3]. The central question thus shifts to assessing their relative contributions to community diversity, distribution, and succession.

Microorganisms are integral to freshwater ecosystems, driving nutrient cycling, organic matter decomposition, pollutant degradation, and energy flow [4]. Understanding their assembly mechanisms across different water habitats is essential for predicting ecosystem responses and informing differentiated management strategies [1]. Recent advances in high-throughput sequencing and ecological modeling have greatly facilitated quantitative analyses of microbial community assembly. Current community assembly theory partitions assembly into five quantifiable processes, including homogeneous selection (HoS), heterogeneous selection (HeS), homogenizing dispersal (HD), dispersal limitation (DL), and ecological drift (DR), offering a unified basis for deciphering the mechanisms that maintain microbial diversity [5]. Stegen et al. (2013, 2015) pioneered a null-model approach based on phylogenetic β-diversity (βNTI) and taxonomic β-diversity (RC) to statistically disentangle selection, dispersal, and drift at the community level [5,6]. Most existing assembly studies rely on the null-model approaches to calculate assembly metrics based on the average phylogenetic or taxonomic turnover across the entire community. A fundamental limitation of these approaches is that they treat all microbial lineages as a homogeneous group and average their responses, which can mask substantial variation among phylogenetic lineages. Different microbial lineages within the same community may respond differently to environmental filters and dispersal barriers, yet whole-community metrics average over these differences, potentially masking important ecological variation [7]. This limitation motivated the development of iCAMP (Community Assembly Mechanisms by Phylogenetic-bin-based null model analysis), which shifts the analytical unit from the whole community to phylogenetic bins [7]. This method assigns species to bins according to their phylogenetic relatedness, performs null modeling for each bin, and aggregates results weighted by bin relative abundance, thereby capturing process differences among microbial lineages more accurately [7,8]. Simulation tests have demonstrated that iCAMP outperforms traditional approaches in accuracy, precision, and sensitivity [7]. The iCAMP framework has since been applied across diverse ecosystems, including marine [9], urban river [10,11], soil [12], and constructed wetland systems [13], and proven wide applicability and superiority.

The assembly of microbial communities in diverse freshwater habitats has received growing research interest. For urban rivers, Liao et al. (2025) examined microbial communities across nine representative Chinese rivers and reported that DL and DR jointly dominated community assembly, promoting spatial heterogeneity [14]. Similarly, Cai et al. (2025) found DL to be the predominant process for both bacteria and micro-eukaryotes throughout all seasons in the Xiangjiang River [15]. In river–lake systems, Wang et al. (2025) investigated the middle Yangtze reaches together with Dongting Lake and Poyang Lake, revealing that stochastic processes, particularly DL, governed sediment microbial assembly, whereas deterministic processes exerted stronger influences on planktonic bacteria and archaea in the lakes [16]. For aquaculture environments, Mao et al. (2025) compared assembly mechanisms of shared and unique bacterioplankton across three typical freshwater aquaculture ponds (crayfish, giant prawn, and grass carp), identifying DL and HoS as the main ecological forces driving community variation, with dynamic interactions involving temperature, selenium, and antibiotics [17]. Bick et al. (2025) further revealed through microcosm experiments that nutrient availability and community size jointly determine the degree of stochasticity in freshwater bacterial community assembly [18].

While previous work has laid an important foundation for understanding freshwater microbial assembly, most studies have been confined to single habitat types, such as urban rivers, lakes, or aquaculture ponds, and thus lack systematic multi-habitat comparisons under comparable temporal scales. Nutrient loading, hydrological connectivity, and human interference vary markedly between artificial (e.g., aquaculture ponds) and natural waters (rivers, lakes), but how these factors influence community assembly has yet to be tested directly. Furthermore, most studies have predominantly examined assembly at the whole-community level, with limited attention to whether individual lineages shift their assembly strategies among habitats. The iCAMP framework, with its phylogenetic-bin resolution, provides an effective tool to investigate this lineage-level plasticity.

Therefore, we selected four freshwater systems representing distinct hydrological and management regimes, including two aquaculture ponds, one enclosed urban lake, and one flowing river, which serves as occasional water replenishment source for the other three water bodies, and conducted monthly sampling over one year. Using 16S rRNA gene high-throughput sequencing combined with the iCAMP framework, this study aimed to address three specific questions: (1) How do different assembly processes change among water habitats and seasons? (2) Does the same phylogenetic lineage exhibit plasticity in its assembly strategy across different habitats? (3) Which environmental factors are the most important in driving each ecological process? Our findings are expected to offer new insights into freshwater microbial community assembly along a gradient of anthropogenic disturbance, and to inform differentiated microbial management strategies for contrasting aquatic systems.

## 2. Materials and Methods

### 2.1. Study Area

Four freshwater systems representing distinct hydrological and management regimes, including two aquaculture ponds, one enclosed urban lake, and one flowing river, were selected for this study. The four freshwater systems were all located near or in Hunan Normal University, in Yuelu District, Changsha City, Hunan Province, south-central China, with straight-line distances of less than 1 km between any two water bodies. The two aquaculture ponds (WC01: 28°11′23′′ N, 112°56′47′′ E, WC02: 28°11′25′′ N, 112°56′47′′ E) were situated at the Genetic Breeding Center of Common Carp and Crucian Carp of Agriculture Ministry (Hunan Normal University), with surface areas of 800 m^2^ and mean depth of 1.5 m. The aquaculture ponds were stocked primarily with common carp, grass carp, and bream, etc., receiving daily feeding throughout the year. Taozi Lake (TZ, 28°11′15″ N, 112°56′50″ E), adjacent to the university campus, is a small urban closed lake, covered 0.08 km with an average depth of 1.5 m. The lake has no natural inflow or outflow streams; its water supply is maintained primarily through atmospheric precipitation and occasional replenishment from the Xiangjiang River. Since 2010, all domestic sewage inflow into the lake has been diverted through a water quality restoration project [19]. The Xiangjiang River (XJ: 28°11′10″ N, 112°57′05″ E), the largest river in Hunan Province, flows northward through five cities before discharging into Dongting Lake [20]; its Changsha section, near Hunan Normal University, was selected for sampling to maintain climatic consistency across all sites.

### 2.2. Sampling and Environmental Factors

Surface (0.5 m in depth) water samples (2 L) were collected monthly from November 2023 to October 2024 from the four water bodies. At each sampling occasion, three replicate water samples were collected from three fixed sampling positions within each water body (approximately 10 m apart) and pooled into one composite sample per water body to reduce within-site spatial heterogeneity [21]. Sampling was conducted between 9:00 and 11:00 a.m. on days without heavy rainfall within the preceding 48 h to minimize the effects of precipitation. Water temperature (T), dissolved oxygen (DO), pH, oxidation-reduction potential (ORP), electricity conductivity (EC), and nephelometric turbidity units (NTU) were determined in situ using a multi-parameter water quality sonde (Aquaread AP-2000, Broadstairs, UK). Nutrient and water quality parameters, including total nitrogen (TN), total phosphorus (TP), ammonium (NH_4_-N), nitrate (NO_3_-N), phosphate (PO_4_-P), chemical oxygen demand (COD), and Chlorophyll a (Chl-a, a proxy for total phytoplankton biomass) were measured in the laboratory according to established standard methods [22]. For molecular analysis, 200~250 mL of fresh water from each sample was filtered through a 0.2 μm polycarbonate membrane (Merck Millipore Ltd., Carrigtwohill, Ireland), and the filters were preserved at −80 °C until DNA extraction.

### 2.3. DNA Extraction, PCR and Sequencing

DNAs on the filters were extracted using the PowerWater DNA Isolation Kit (MoBio Laboratories, Carlsbad, CA, USA) following the manufacturers’ instructions. DNA integrity was assessed by agarose gel electrophoresis, with clear bands and no visible smearing as the acceptance criterion. DNA concentration and purity were measured using a Qubit 3.0 Spectrophotometer (Thermo Fisher, Waltham, MA, USA), requiring a minimum concentration of 25 ng/μL and a total amount of at least 2 μg. The DNAs were then sent to Genesky Biotechnologies Inc., (Shanghai, China) for a full-length 16S rDNA amplicon sequencing. Briefly, the full-length 16S rRNA gene was amplified using the universal prokaryote primers 27F ((5′-AGRGTTYGATYMTGGCTCAG-3′) and 1492R (5′-RGYTACCTTGTTACGACTT-3′) [23] tailed with10-bp sample-specific barcode sequences. The purified long 16S rRNA gene fragments were then pooled for subsequent library construction with the PacBio SMRTbell prep kit 3.0 (PacBio, Menlo Park, CA, USA), following the manufacturer’s instructions. The resulting SMRTbell libraries were purified and subjected to quality control, and sequencing was performed on the PacBio Revio platform (v13.3).

### 2.4. Sequencing Data Processing and Analysis

Raw FASTQ reads were first filtered by quality and length using Chopper v0.12.0 to eliminate low-quality sequences and those with undesired lengths [24]. The filtering parameters were set as --quality 10, --minlength 1000 and --maxlength 1800. Subsequent processing was carried out in QIIME2 (version 2023.2) [25], followed by error correction, primer trimming, and chimera removal via the DADA2 plugin to generate amplicon sequence variants (ASVs). Finally, high-quality ASV feature tables and representative sequences were obtained. Taxonomic assignment of ASVs was performed using the DADA2 assignTaxonomy function against the SILVA database (version 138.2) [26], using a confidence threshold of 0.5. Chloroplast and unassigned sequences were discarded, and ASVs detected in only one sample (singletons) were also excluded from further analysis.

The raw sequence data reported in this paper have been deposited in the Genome Sequence Archive [27] in National Genomics Data Center [28], China National Center for Bioinformation/Beijing Institute of Genomics, Chinese Academy of Sciences (GSA: CRA047929; BioProject: PRJCA071262) that are publicly accessible at https://ngdc.cncb.ac.cn/gsa (accessed on 9 September 2026).

### 2.5. Statistical Analysis

To normalize sequencing depth, the ASV table was rarefied to 38,065 reads per sample, corresponding to the minimum depth across all 48 samples. Species richness (the number of observed ASVs) and the Shannon index were calculated as alpha diversity index. Community dissimilarity was visualized via principal coordinate analysis (PCoA) based on Bray–Curtis distances. PERMANOVA (999 permutations restricted within each water body) was used to test the effects of water type and season on bacterial community structure, and principal component analysis (PCA) was applied to compare environmental characteristics among habitats.

The iCAMP framework was employed to quantify the relative contributions of stochastic (homogenizing dispersal, dispersal limitation and drift) and deterministic (homogeneous selection and variable selection) processes to community assembly [7]. Recognizing that different taxa within the same community may be governed by distinct processes, iCAMP first assigns taxa to phylogenetic bins and then identifies the assembly processes for each bin. The ecological processes assignment follows a previously established procedure [6] based on the values of the net relatedness index (βNRI) and the Bray–Curtis-based Raup-Crick metric incorporating species’ relative abundances (RC_bray_). Briefly, for each bin, the fraction of pairwise turnovers with βNRI > 1.96 or <−1.96 indicate heterogeneous or homogeneous selection, respectively. For the fraction of turnovers with non-significant phylogenetic dissimilarity (−2 ≤ βNRI ≤ 2), RC_bray_ values > 0.95 or <−0.95 indicate dispersal limitation or homogenizing dispersal, respectively. The remaining fraction (−2 ≤ βNRI ≤ 2 and −0.95 ≤ RC_bray_ ≤ 0.95) is attributed to drift. The iCAMP analysis was run with the following parameter settings: the minimum number of ASVs per bin (nmin) was set to 24; the number of null-model permutations was set to 999; βNRI was calculated based on within-bin randomization, while RC_bray_ was estimated based on across-bin randomization. The other settings follow the recommendations of the original iCAMP publication [7]. The relative importance of each process in each bin was summarized and weighted by bin-relative abundances to derive taxa to community-level estimates, including relative importance of different ecological processes in each pairwise comparison (P_tuv_), relative importance of different processes in governing each bin/taxa (P_tk_), and bin/taxa relative contribution to each process (BRP_tk_). The significance of difference in relative importance of ecological processes among water types and seasons was calculated by two-way ANOVAs. To evaluate the effect of water type on each ecological process, we calculated Cohen’s d as the difference in means between two water types divided by the pooled standard deviation. Effect sizes were interpreted as large (|d| > 0.8), medium (0.5 < |d| ≤ 0.8), small (0.2 < |d| ≤ 0.5), or negligible (|d| ≤ 0.2).

Relationships between ecological processes and environmental variables were examined using Mantel tests and distance-based multivariate regression (MRM). The best MRM model was selected by forward stepwise selection based on Akaike information criterion (AIC). For each environmental factor (e.g., temperature), both the absolute difference (e.g., |Ti − Tj|, where i and j represent samples) and the mean ([Ti + Tj]/2) between pairwise samples were calculated for correlation with the relative importance of each process. All statistical analyses and visualization were performed in the R environment (v4.3.2) using the following packages: iCAMP 1.5.12, vegan 2.6.4, ape 5.7.1, ecodist 2.1.3, corrplot 0.92, psych 2.3.9, tidyr 1.3.0, VennDiagram 1.7.3, ggalluvial 0.12.5, and ggplot2 3.4.4.

## 3. Results

### 3.1. Bacterial Diversity and Composition Across Contrasting Water Habitats

**Figure 1:**
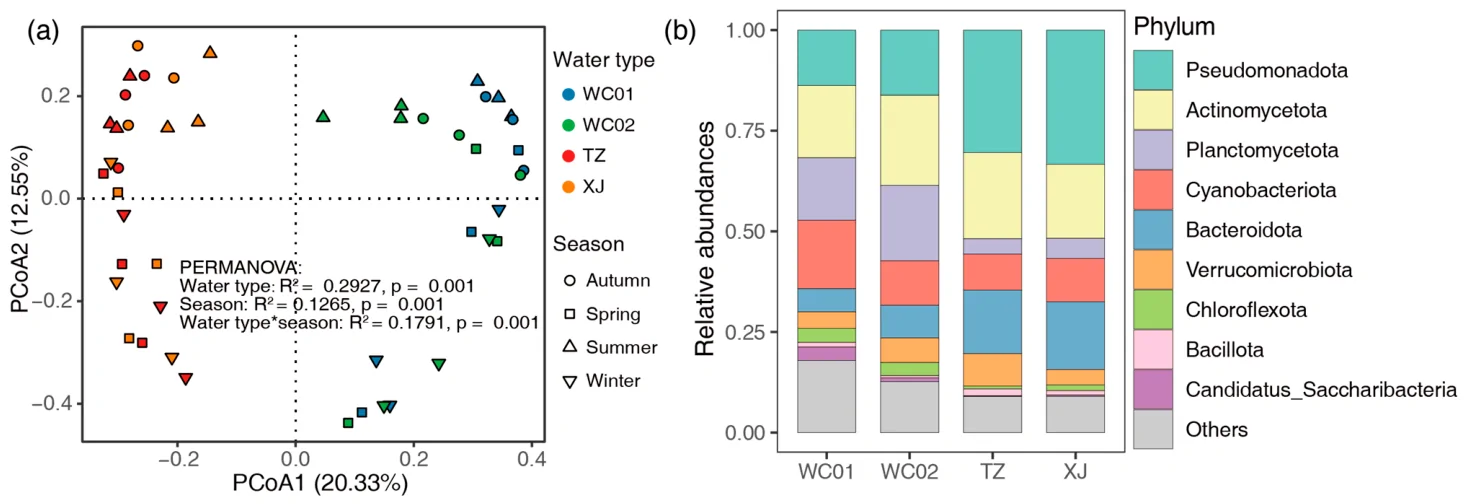
Bacterial diversity and composition across different freshwater habitats. (**a**) Principal coordinate analysis (PCoA) showing the community variation patterns among water types and seasons. (**b**) Relative abundances of the dominant phyla among different water bodies. Taxa with mean relative abundance <2% across all samples were grouped into ‘Others’.

Based on 16S rRNA gene high-throughput sequencing data, alpha diversity indices, including observed ASV richness and Shannon index, were calculated for each sample, which revealed significant differences among water bodies (*p* < 0.05, Figure S1). The flowing river XJ exhibited the highest richness (mean = 3145) and Shannon diversity (mean = 6.84), followed by the two aquaculture ponds (WC01: richness = 2278, Shannon = 6.49; WC02: richness = 1957, Shannon = 6.20), while the urban lake TZ showed lower diversity (richness = 1691, Shannon = 5.79). PERMANOVA tests were used to assess the effects of water type and season on bacterial community structure. Water type emerged as the primary driver of community variation (R^2^ = 0.2927, *p* < 0.001), with season (R^2^ = 0.1265, *p* < 0.001) and their interaction (R^2^ = 0.1791, *p* < 0.001) also showing significant but considerably weaker influences. Consistently, PCoA revealed that communities from the two artificial aquaculture ponds (WC01 and WC02) formed a cluster, whereas those from the urban lake (TZ) and the flowing river (XJ) grouped together as a distinct natural-water cluster, clearly separating artificial from natural water habitats along the first axis (Figure 1a). Taxonomic composition of the bacterial community further corroborated this separation (Figure 1b). In the two aquaculture ponds, the bacterial community was dominated by *Actinomycetota* (mean relative abundance 20.2%), *Planctomycetota* (mean 17.1%), *Pseudomonadota* (mean 15.0%), and *Cyanobacteriota* (mean 14.0%). In contrast, the natural water habitats exhibited a different compositional pattern: *Pseudomonadota* became the most abundant phylum, rising markedly to 31.9%, followed by *Actinomycetota* (mean 19.9%), *Bacteroidota* (mean 16.3%, substantially enriched), and *Cyanobacteriota* (mean 9.8%, slightly reduced).

### 3.2. Community Assembly Patterns Across Water Types and Season

**Figure 2:**
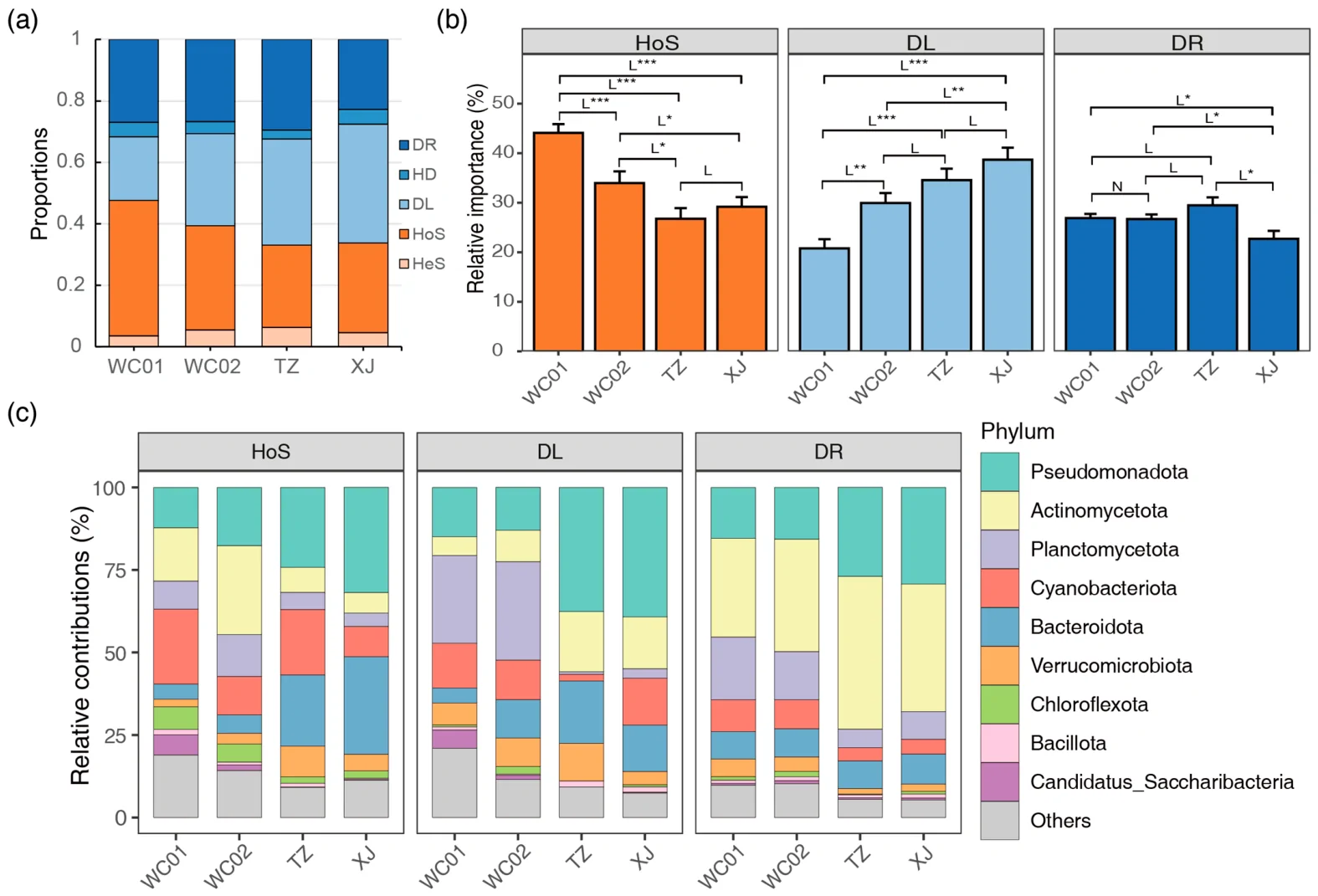
(**a**) Relative importance of different ecological assembly processes across different water bodies. HoS, homogeneous selection; HeS, heterogeneous selection; DL, dispersal limitation; HD, homogenizing dispersal; DR, drift. (**b**) Changes in HoS, DL, and DR across different water bodies. Significance levels: *** *p* < 0.001, ** *p* < 0.01, * *p* < 0.05. L, M, S and N indicated large, medium, small and negligible effect sizes of water type based on Cohen’s d. (**c**) Relative contributions of different phyla to HoS, DL, and DR across different water bodies. Taxa with mean relative abundance < 2% across all samples were grouped into ‘Others’.

Using the iCAMP null-model framework, we quantified the relative contributions of various assembly processes across different water bodies. The results indicated that homogeneous selection (HoS, 33.5%), dispersal limitation (DL, 31.0%), and drift (DR, 26.5%) were the dominant process in bacterial community assembly, with a combined importance of 91.0% on average across all samples. Heterogeneity selection (HeS) and homogeneous dispersal (HD) made up only a small fraction (Figure 2a). We therefore concentrated further analyses on these three major processes. To test the effects of water type, season, and their interaction on the relative importances of the three dominant assembly processes (HoS, DL, and DR), we performed two-way ANOVAs. Water type emerged as the only factor with a significant influence on all three processes (*p* < 0.05), whereas neither season nor their interaction reached significance (*p* > 0.05, Table 1). Specifically, the relative importance of HoS in the two aquaculture ponds (WC01 and WC02) were significantly higher than those in the natural water bodies (XJ and TZ). In the flowing river XJ, DR contributed the least among the three processes, while DL contributed the most (Figure 2b). These findings suggested that habitat filtering acted as a primary stabilizing force in regulating community assembly.

We further examined the core taxa responsible for each assembly process in different water types based on the weighted BRP_tk_ contribution of iCAMP bins. The results indicated that the core taxa responsible for the same assembly process differed markedly among water types, with the primary divergence occurring between the two aquaculture ponds (WC01 and WC02) and the natural water bodies (XJ and TZ, Figure 2c). In WC01 and WC02, HoS was mainly contributed by *Actinomycetota* and *Cyanobacteriota*, with additional substantial contributions from *Planctomycetota*, *Chloroflexota*, and *Candidatus Saccharibacteria*—all showing higher contributions than in XJ and TZ. By contrast, in XJ and TZ, HoS was mainly contributed by *Pseudomonadota* and *Bacteroidota*. For DL, *Planctomycetota* was the primary contributor in the aquaculture ponds, whereas *Pseudomonadota* dominated DL in XJ and TZ. For DR, *Actinomycetota* served as the dominant contributor across all four water bodies.

### 3.3. Assembly Mechanisms of Different Phylogenetic Groups

**Figure 3:**
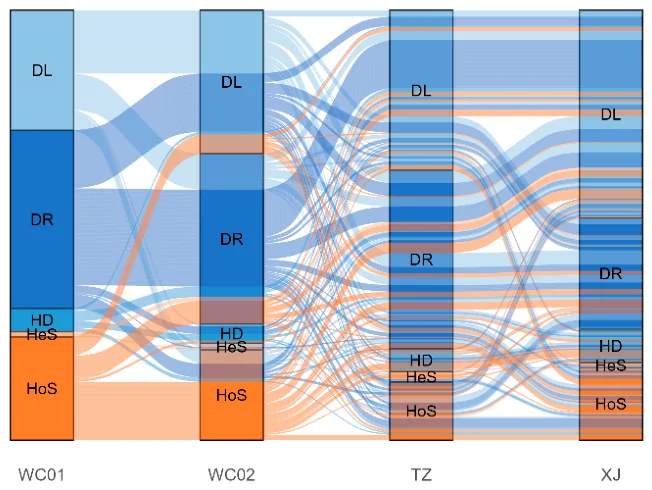
Sankey plots showing the changes in the dominant assembly process of each bin across different water bodies. Each flowing curve in the plot represents a bin. HoS, homogeneous selection; HeS, heterogeneous selection; DL, dispersal limitation; HD, homogenizing dispersal; DR, drift.

We detected a total of 39,047 ASVs in all samples, which were assigned to 338 phylogenetic bins through the iCAMP binning system. The dominant assembly process within the same bin shifted markedly among water bodies, suggesting considerable habitat-dependent plasticity in assembly process of bacterial phylogenetic groups. The Sankey diagram illustrated these transition patterns across the four water bodies, showing that most bins switched their dominant processes as habitat changed (Figure 3). The Venn diagrams summarized the numbers of shared and unique bins dominated by HoS, DL, or DR across the water bodies (Figure S1). In all four water bodies, 162, 273, and 283 bins were dominated by HoS, DL, and DR, respectively. However, bins shared by all four water bodies with the same dominant process accounted for only about 2% of the total for each process (Figure S2). However, it’s worth noting that the number of shared bins dominated by the same processes between the two aquaculture ponds was usually the highest. These results implied that habitat type served as a primary determinant of lineage assembly strategy, and that similar environmental filtering within the same water type led to similar assembly outcomes.

**Figure 4:**
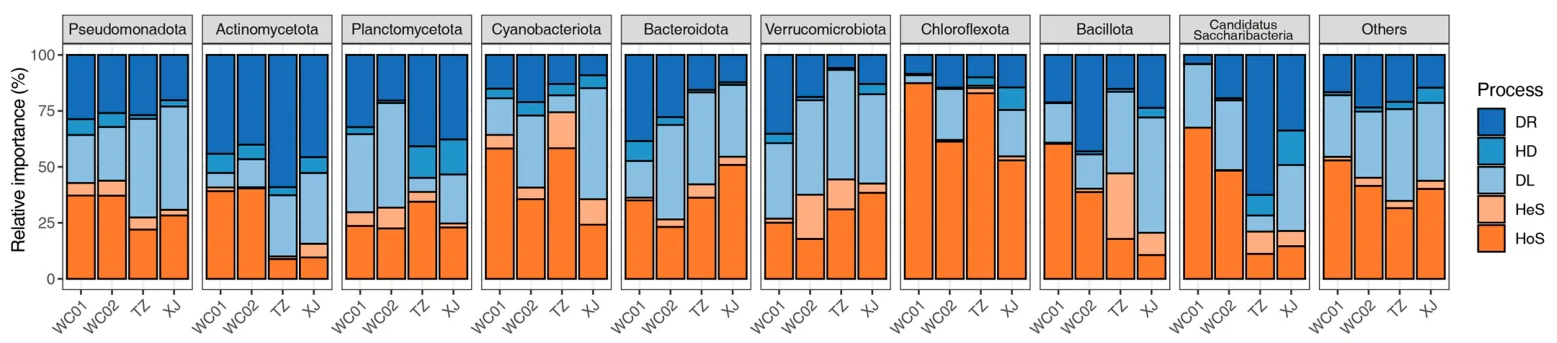
Relative importances of various ecological processes in governing each phylum across different water bodies. HoS, homogeneous selection; HeS, heterogeneous selection; DL, dispersal limitation; HD, homogenizing dispersal; DR, drift. Taxa with mean relative abundance < 2% across all samples were grouped into ‘Others’.

The weighted P_tk_ summary at the phylum level further illustrated the plasticity of lineage assembly across habitats (Figure 4). Only two phyla—*Chloroflexota* and *Cyanobacteriota*—showed consistent HoS dominance across all four sites; the dominant processes for most other phyla shifted notably with water type. Specifically, in the two aquaculture ponds, HoS exerted a substantially stronger influence on *Actinomycetota*, *Bacillota*, *Pseudomonadota*, and *Candidatus Saccharimonadia* than in the natural water bodies (TZ and XJ). In those natural habitats, by contrast, these taxa were largely governed by stochastic processes, with *Actinomycetota* and *Candidatus Saccharimonadia* being dominated by DR, and *Pseudomonadota* and *Bacillota* being dominated by DL.

### 3.4. Environmental Factors Influencing Bacterial Assembly Processes

PCA of environmental variables (Table S1) revealed distinct separation of the four water bodies along physicochemical gradients, especially between the two aquaculture ponds (WC01 and WC02) and two natural water bodies (TZ and XJ). The aquaculture ponds were characterized by high nitrogen, phosphorus, and Chl-a levels, whereas XJ and TZ exhibited higher ORP and DO values (Figure S3).

**Figure 5:**
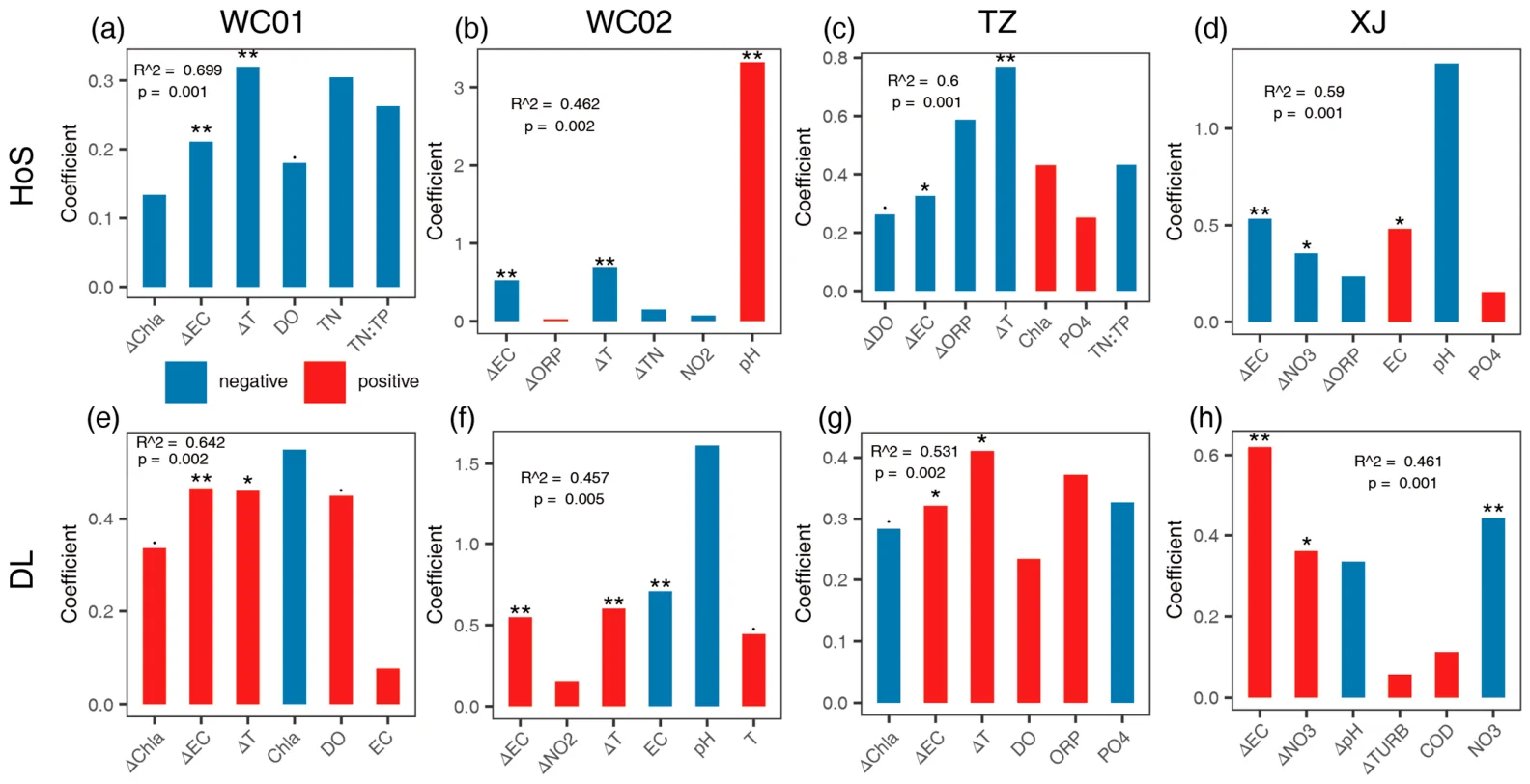
Results of multiple regression on distance matrix (MRM) showing the effects of environmental factors on homogeneous selection (HoS, upper panel) and dispersal limitation (DL, lower panel) in WC01 (**a**,**e**), WC02 (**b**,**f**), TZ (**c**,**g**) and XJ (**d**,**h**). The difference (with a Δ before the name) and mean (without Δ) of each environmental factor between pairwise samples were used for the analysis. Significance levels: ** *p* < 0.01, * *p* < 0.05, · *p* < 0.1.

We further applied Mantel tests and MRM to examine the environmental factors associated with the three ecological assembly processes (HoS, DL, and DR). The mantel tests indicated that HoS and DL showed similar relationships with environmental factors but in opposite directions across all water bodies (Figure S3). Across all water bodies, temperature and conductivity difference were consistently significantly correlated with HoS and DL. In addition, in the two aquaculture ponds, HoS and DL were mostly significantly correlated with nutrient related factors, including nitrogen, phosphorus and Chl-a; however, in TZ and XJ, they were also strongly correlated with ORP and turbidity (Figure S4). The MRM analysis further revealed that, across all water bodies, temperature and conductivity difference acted as core factors correlated with both HoS and DL (Figure 5). Beyond these common factors, habitat-specific factors also emerged. In the two aquaculture ponds, nitrogen- and phosphorus-related variables (TN, TN:TP, NO_2_^−^) and Chl-a were the primary factors positively correlated with HoS (Figure 5a,b). In TZ and XJ, DO, turbidity, and ORP were the key factors associated with DL variation (Figure 5g,h). For DR, environmental forcing was weak (R^2^ < 0.4) in the aquaculture systems and largely nonspecific in the natural sites, where influences from ORP, DO, and pH were detected (Figure S5).

## 4. Discussion

### 4.1. Water Type Is the Dominant Factor Shaping Bacterial Community Diversity

In this study, we selected four typical freshwater habitats, including two aquaculture ponds (WC01, WC02), an urban lake (TZ), and a flowing river (XJ), and conducted monthly sampling over 12 months to examine the spatiotemporal dynamics of bacterial communities and their driving factors. Many studies have reported that temperature-driven seasonal variation is a major force shaping bacterial communities [10,22]. In contrast, in the present study, PERMANOVA results identified water type as the dominant factor explaining community variation, with season and their interactions showing significant but considerably weaker effects. This was visually supported by PCoA, which clearly separated all samples into two distinct clusters—artificial aquaculture ponds (WC01 and WC02) versus natural water bodies (TZ and XJ)—reinforcing the long-term filtering role of habitat heterogeneity [29,30]. This discrepancy may arise from the fact that most previous studies focused on single water bodies, thus, their findings primarily reflected local temporal patterns. In the present study, the inclusion of contrasting water habitat types (artificial vs. natural, lentic vs. lotic) revealed that habitat heterogeneity exerts a much stronger regulatory factor on community variation than short-term seasonal disturbances [30].

Taxonomic compositional analysis further supported the effect of habitat filtering. In the two eutrophic aquaculture ponds (WC01, WC02), the bacterial communities were dominated by *Actinomycetota*, *Planctomycetota*, and *Cyanobacteriota*. However, in the two natural water bodies (TZ and XJ), significant enrichment in *Pseudomonadota* and *Bacteroidota* was observed. This pattern aligns with previous studies in rivers and lakes, which have shown that artificial nutrient loading and natural hydrological conditions impose distinct environmental filters that select for bacterial groups with different nutrient tolerance spectra [31]. In addition, traditional community diversity analyses, which often describe only compositional variations, are limited in addressing the underlying assembly mechanisms [32]. Our results indicate that the pronounced community variation across water types is not stochastic; rather, it arises from the combined effects of long-term deterministic environmental filtering and stochastic dispersal limitation. The observed community variations essentially reflect how different phylogenetic lineages respond to habitat-specific filtering and dispersal constraints. Consequently, the same bacterial phylum can exhibit entirely different assembly patterns across different water types.

### 4.2. Bacterial Communities from Different Water Bodies Show Distinct Assembly Patterns

Based on the iCAMP null-model framework, we quantified the relative importance of various ecological processes in bacterial community assembly. At the whole-community level, homogeneous selection (HoS, 33.5%), dispersal limitation (DL, 31.0%), and drift (DR, 26.5%) were the dominant processes, whereas heterogenous selection (HeS) and homogenizing dispersal (HD) contributed only marginally. This pattern is consistent with recent studies from diverse aquatic systems, including oceans, rivers, lakes, and aquaculture ponds [17,33,34,35]. The agreement across such varied habitats suggests that HoS and DL may represent a general feature of aquatic microbial assembly. Generally, surface water habitats exhibit moderate environmental heterogeneity, with few extreme patches, so HeS is seldom as strong as in groundwater systems [8]; consequently, HoS prevails in most surface waters [10,36]. Concurrently, the inherent connectivity of water bodies prevents complete isolation, limiting the occurrence of large-scale homogeneous diffusion [34].

Two-way ANOVAs further confirmed that water type, rather than season or their interaction, significantly affected the relative contributions of HoS, DL, and DR. This suggests that, over the one-year study period, habitat filtering acted as the primary driver of community assembly, outweighing seasonal fluctuations, which was consistent with previous observations where seasonal disturbances within the year only induced short-term local shifts and did not alter the annual average assembly pattern [37]. We acknowledge that with only four individual water bodies, water type is partially confounded with site identity, and caution should be exercised when generalizing these findings to other freshwater systems without further validation. Specifically, HoS was significantly stronger in the two aquaculture ponds (WC01, WC02) than in the two natural water bodies (XJ, TZ). This aligns with the environmental conditions of the aquaculture systems, which sustain a homogeneously eutrophic state, with high nitrogen, phosphorus, and Chl-a levels, due to consistent feeding, fertilization, and water management [17,38]. Such strong eutrophic pressure reinforces deterministic HoS. Similarly, Mao et al. (2025) also reported that bacterial assembly in aquaculture ponds was governed by HoS and DL, with environmental factors (e.g., temperature, selenium, antibiotics) dynamically interacting with these processes [17]. Consistently, the lower diversity (Figure S1) in aquaculture ponds aligns with stronger HoS filtering the community toward a smaller set of tolerant taxa. In addition, the relative importance of DL was significantly increased in the flowing river XJ, reflecting a distinct diffusion-selection balance in lotic systems. This can be ascribed to the high flowability and low hydraulic retention time, which promote frequent species dispersal, while spatial and temporal isolation and upstream–downstream environmental gradients simultaneously create effective dispersal barriers [37]. In contrast, in the static water bodies (WC01, WC02, and TZ), weaker hydraulic connectivity and reduced spatial isolation led to higher DR proportions, indicating enhanced stochastic population fluctuations in static water bodies. These observations highlight hydraulic connectivity as a key habitat attribute modulating the relative importance of various stochastic assembly processes [31].

### 4.3. Bin-Level Analysis Reveals High Assembly Plasticity of Bacterial Lineages

Traditional null models (e.g., QPEN) are based on the pairwise turnovers of the whole communities, which provide only an estimate of ecological processes of the entire community [5,39]. However, microbial taxa differ in their responses to environmental factors, as well as in their diversification rates, dispersal capacities, and sensitivity to drift [7]. Thus, estimates of ecological processes derived from whole-community metrics inevitably mask variation in assembly strategies among lineages. The iCAMP framework, which first partitions taxa into phylogenetic bins and then quantifies the processes governing each bin, overcomes this limitation and provides a more resolved view at the bin level [7]. In our study, the whole-community level results showed that HoS, DL, and DR were the dominant processes in all four water habitats, which might suggest convergent assembly across habitats. However, the bin-level results revealed that the majority of bins shifted their assembly processes across water bodies, demonstrating that the whole-community values concealed extensive lineage-specific variation in assembly strategies. These findings suggested that freshwater bacteria may exhibit habitat-dependent assembly plasticity: homogeneous habitats drive convergence of lineage assembly strategies through similar environmental filtering and dispersal conditions, whereas heterogeneous habitats force lineages to adopt alternative assembly strategies [40].

Specifically, *Chloroflexota* and *Cyanobacteriota* were consistently governed by HoS across all water habitats. This pattern suggested that these two lineages might possess relatively broad ecological niches and they remain under HoS control as long as local environmental conditions are homogeneous, whether in eutrophic aquaculture ponds or in natural rivers and lakes. Indeed, *Cyanobacteriota* can utilize low concentrations of nitrogen and phosphorus and tolerate temperature fluctuations [41], while most *Chloroflexota* are oligotrophic degraders and are relatively insensitive to ionic composition and hydraulic conditions [42]. Consequently, deterministic selection in homogeneous environments prevails for these taxa regardless of habitat type, with no significant transition in assembly strategy; they thus represent low-plasticity lineages. In contrast, most other microbial groups exhibited high assembly plasticity. For example, *Actinomycetota*, *Bacillota*, *Pseudomonadota*, and *Candidatus Saccharimonadia* were governed by HoS in the eutrophic aquaculture ponds (WC01, WC02), but shifted to stochastic processes (DL or DR) in the flowing river XJ and the enclosed lake TZ. This lineage-level variation suggested that the same taxonomic group may be governed by different assembly processes across habitats, which could reflect divergent survival strategies [43] but cannot be confirmed as active strategy shifts from observational data alone. This might be an important reason why the same assembly process is contributed by significantly different categories in different water bodies (Figure 2c). *Pseudomonadota*, for instance, contributed substantially to both HoS and DL in natural waters, but played a much smaller role in the eutrophic aquaculture ponds. This pattern suggests that in homogeneous eutrophic environments, taxa assembly is primarily driven by deterministic selection, whereas in oligotrophic or hydrologically fluctuating conditions, stochastic processes tend to play a more prominent role. Similarly, environmental selection has also been reported to be more important in lentic lakes than in lotic streams [37].

The widespread assembly plasticity at the bin/taxa level observed in this study challenges the traditional assumption of stable species ecological niches [44]. Microorganisms belonging to the same taxonomic group can assume distinctly different ecological roles depending on environmental conditions—sometimes shaped primarily by deterministic selection, and other times by stochastic processes. This implies that predicting microbial community responses to environmental change requires moving beyond community level estimations and focusing on finer biological organization levels [7,8,45,46]. Furthermore, assembly patterns derived from single habitat are unlikely to be generalizable across habitats. Many previous freshwater investigations sampled only individual lake or watershed and thus failed to detect the phylogenetic plasticity revealed here. By incorporating artificial versus natural and static versus flowing water types as orthogonal contrasts, our study highlights the importance of multi-habitat comparisons for revealing lineage-dependent assembly flexibility.

### 4.4. The Key Environmental Factors Driving Ecological Processes Differ Across Water Types

Temperature and conductivity difference were consistently correlated with both HoS and DL across all samples, yet with opposite regression directions between HoS and DL. Greater differences in temperature and ionic conditions between two samples led to reduced HoS and increased DL, whereas homogeneous conditions favored HoS over DL. Temperature directly affects the physiological limits for microbial activity [47], while EC not only reflects overall nutrient and ion levels but also serves as an indicator of hydrologic process [48]. Together, these two variables greatly determine the degree of environmental homogeneity across samples, effectively acting as key drivers in freshwater community assembly [49]. In the eutrophic aquaculture ponds, internal differences were small and annual environmental homogeneity remained high, allowing HoS to persist as the dominant force. In contrast, the river and lake sites exhibited greater monthly fluctuations in temperature and nutrient gradients, where enhanced environmental heterogeneity promoted DL [50].

However, besides temperature and conductivity difference, the results of MRM and Mantel tests also identified distinct environmental drivers for ecological processes in different water bodies. In the two aquaculture ponds, nitrogen and phosphorus nutrients (TN, TN:TP, NO_2_^−^) and Chl-a emerged as important factors positively correlated with HoS, confirming that eutrophication promotes homogeneous selection [51,52]. In contrast, DO, turbidity, and ORP were the key factors associated with DL in the natural water bodies (TZ and XJ). This divergence reflects the distinct forces shaping community assembly: nutrient inputs from artificial management dominate deterministic selection in aquaculture systems, whereas hydrodynamic and redox conditions primarily control dispersal limitation in natural waters. For DR, no specific environmental drivers were identified in the present study; only minor influences from pH and temperature were detected. This pattern aligns with the neutral theory that drift arises from random population fluctuations, governed largely by effective population size rather than by specific environmental factors [53]. We note that Mantel tests and MRM identify correlations rather than causal relationships, and some environmental variables (e.g., nutrients and Chl-a) may be collinear; the associations reported here should therefore be interpreted as indicative rather than definitive causal drivers.

These findings on habitat-dependent differentiation of assembly processes have practical implications for freshwater management. In aquaculture ponds, nutrient regulation, particularly nitrogen and phosphorus, could shift deterministic assembly and reshape microbial communities. For urban lakes and rivers, improving hydrological connectivity would alleviate dispersal limitation and promote biotic exchange and homogenization.

Nevertheless, an important caveat of the iCAMP framework, and of null-model approaches in general, is that the homogeneous selection signal, as detected by βNRI values, cannot distinguish between abiotic environmental filtering and biotic interactions such as competition or predation [7,8]. Both types of deterministic forces can generate similar phylogenetic clustering patterns. In our study, the stronger homogeneous selection in aquaculture ponds could reflect not only nutrient-driven abiotic filtering but also intensified biotic interactions in these dense, productive systems. Without experimental manipulation or data on biological interactors (e.g., protists, phages), we cannot disentangle these two components. This limitation should be kept in mind when interpreting the lineage-level assembly patterns reported here.

## 5. Conclusions

In this study, we investigated microbial communities across multiple freshwater habitats and identified homogeneous selection, dispersal limitation, and drift as dominant processes governing bacterial community assembly. The relative importance of these ecological processes varied not only among habitats but also across phylogenetic lineages within the same habitat. Among the four investigated systems, water type emerged as the primary factor regulating assembly patterns, with effects exceeding those of seasonal fluctuations. Furthermore, environmental drivers of assembly processes also showed marked habitat-specific patterns. These findings demonstrate that microbial assembly is heterogeneous across habitat types and taxonomic levels, implying that whole-community and lineage-level analyses are complementary rather than substitutable. Given the pressures of global change and eutrophication, elucidating lineage-specific responses to environmental factors is of both theoretical and practical value, which is essential for predicting ecosystem responses, optimizing aquaculture microbial management, and designing effective restoration strategies for natural waters.

## Figures and Tables

**Table 1:** Significances based on Two-way ANOVAs showing the effects of water type and season on the relative importances of homogeneous selection (HoS), dispersal limitation (DL), and drift (DR).

	HoS	DL	DR
Water type	<0.001 ***	<0.001 ***	0.029 *
Season	0.174	0.799	0.510
Water type * Season	0.144	0.216	0.098

Significance levels: *** *p* < 0.001, * *p* < 0.05.

## Data Availability

The raw sequence data reported in this paper have been deposited in the Genome Sequence Archive in National Genomics Data Center, China National Center for Bioinformation/Beijing Institute of Genomics, Chinese Academy of Sciences (GSA: CRA047929, BioProject: PRJCA071262) that are publicly accessible at https://ngdc.cncb.ac.cn/gsa (accessed on 9 September 2026).

## Supplementary Materials

The following supporting information can be downloaded at: https://www.mdpi.com/article/10.3390/biology15181609/s1, Table S1: Environmental factors; Figure S1: Richness (a) and Shannon index (b) across different water bodies. Figure S2: Venn diagrams show the number of shared and unique bins dominated by homogeneous selection (HoS, a), dispersal limitation (DL, b), and ecological drift (DR, c) across different water bodies; Figure S3: Principal component analysis of environmental factors; Figure S4: Heatmaps based on mantel tests showing the correlations between environmental factors and ecological processes in (a) WC01, (b) WC02, (c) TZ, and (d) XJ. HoS, homogeneous selection; DL, dispersal limitation; DR, drift. The first letter (“d” and “m”) represented the difference (“d”) and mean (“m”) of the environmental factors between pairwise samples. Significance levels: *** *p* < 0.001, ** *p* < 0.01, * *p* < 0.05; Figure S5: Results of multiple regression on distance matrix (MRM) showing the effects of environmental factors on drift (DR) in the four water habitats. The difference (with a Δ before the name) and mean (without Δ) of each environmental factor between pairwise samples were used for the analysis. Significance levels: *** *p* < 0.001, ** *p* < 0.01, * *p* < 0.05.
