# Supplementary material for: Bacterial Community Assembly Patterns Across Distinct Freshwater Habitats
Source: Biology (Basel). 2026 Sep 11;15(18):1609. doi: 10.3390/biology15181609 (PMC13604698; doi:10.3390/biology15181609)
Supplement: Supplementary file 1 [file biology-15-01609-s001.zip › biology-4526351-supplementary.pdf]

Bacterial Community Assembly Patterns across Distinct Freshwater Habitats

Shengnan Li [1,*], Zhe Wang [1], Xinyu Xie [1], Xun Xu [1], Min Wang [1], Ting Yi [1], and Ping Wu [1], Qianhong Gu [1]

[1]Engineering Research Center of Polyploid Fish Reproduction and Breeding of the State Education Ministry, College of Life Sciences, Hunan Normal University, Changsha 410081, Hunan, People's Republic of China

* Correspondence: shnli@hunnu.edu.cn

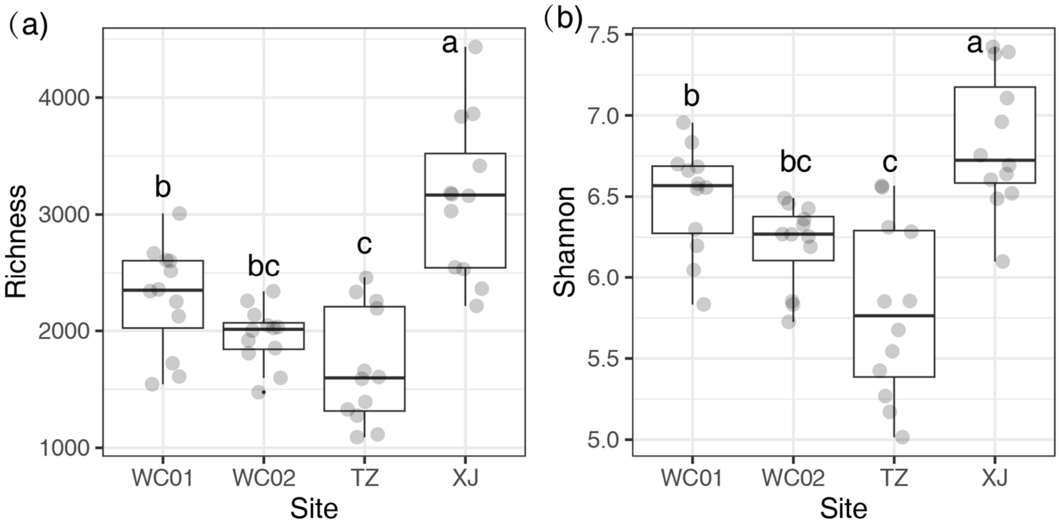

Figure S1. Richness (a) and Shannon index (b) across different water bodies.

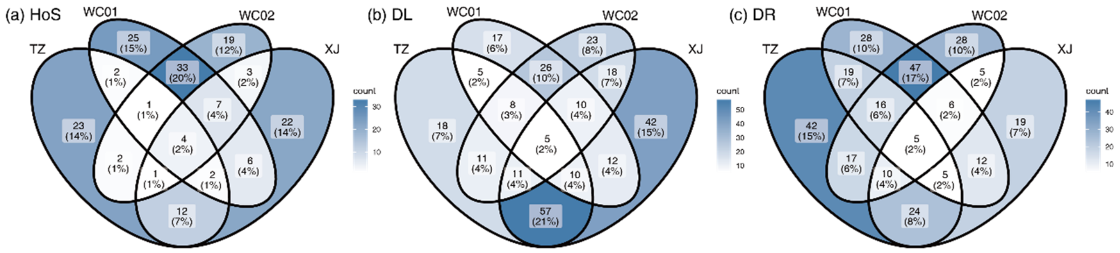

Figure S2. Venn diagrams show the number of shared and unique bins dominated by homogenous selection (HoS, a), dispersal limitation (DL, b), and ecological drift (DR, c) across different water bodies.

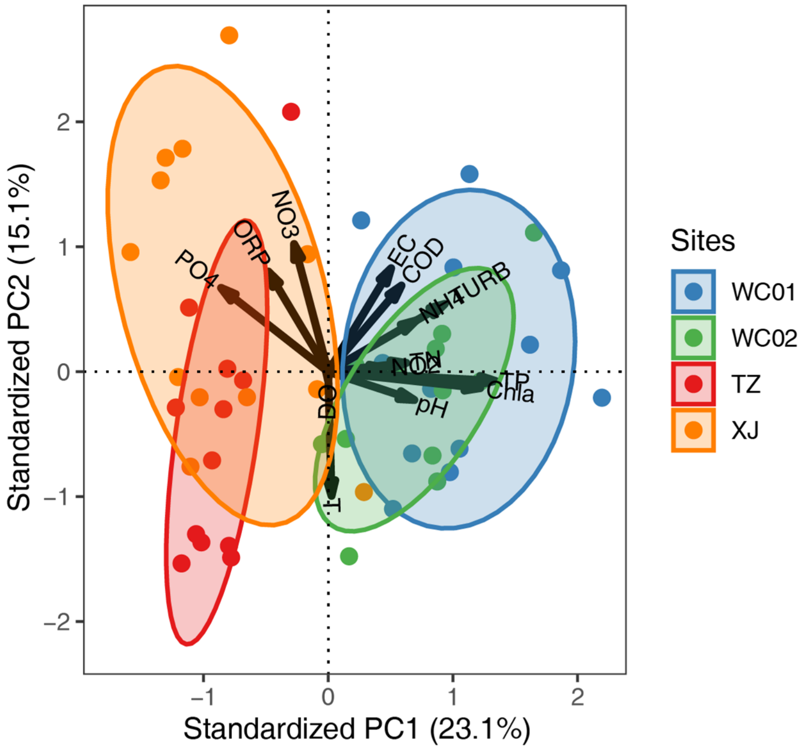

Figure S3. Principal component analysis of environmental factors

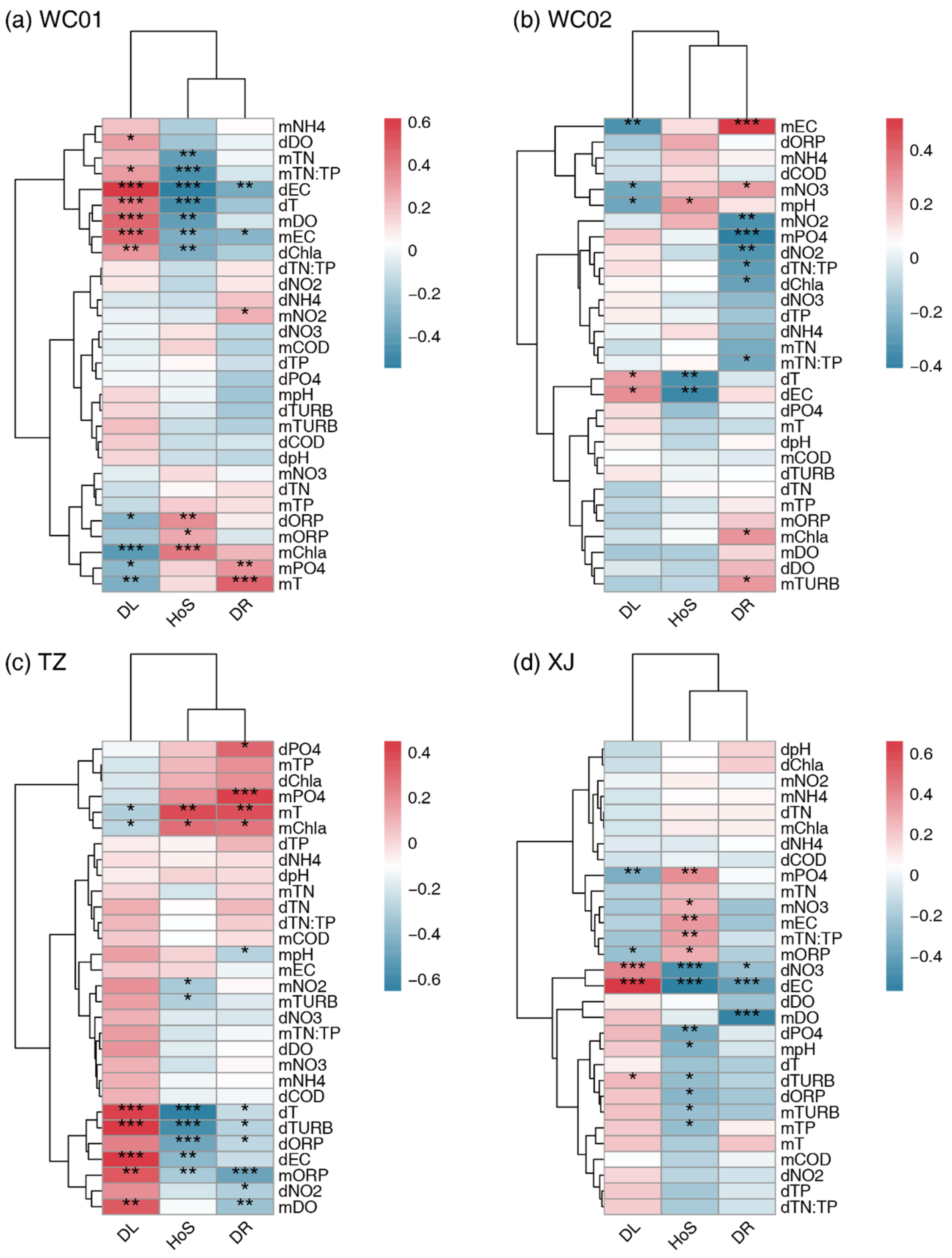

Figure S4. Heatmaps based on mantel tests showing the correlations between environmental factors and ecological processes in (a) WC01, (b) WC02, (c) TZ, and (d) XJ. HoS, homogenous selection; DL, dispersal limitation; DR, drift. The first letter ("d" and "m") represented the difference ("d") and mean ("m") of the environmental factors between pairwise samples. Significance levels: *** $p < 0.001$, ** $p < 0.01$, * $p < 0.5$.

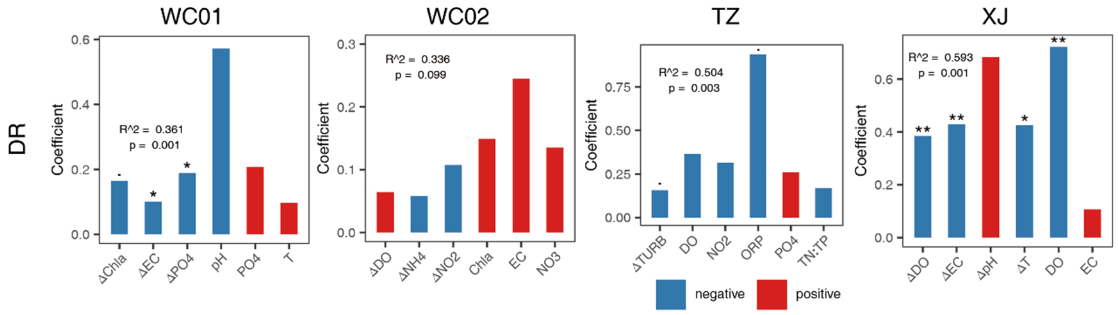

Figure S5. Results of multiple regression on distance matrix (MRM) showing the effects of environmental factors on drift (DR) in the four water habitats. The difference (with a Δ before the name) and mean (without Δ) of each environmental factor between pairwise samples were used for the analysis. Significance levels: *** $p < 0.001$, ** $p < 0.01$, * $p < 0.5$.
